# Retrospective on a decade of machine learning for chemical discovery

**DOI:** 10.1038/s41467-020-18556-9

**Published:** 2020-09-29

**Authors:** O. Anatole von Lilienfeld, Kieron Burke

**Affiliations:** 1 Faculty of Physics, University of Vienna, 1090 Vienna, Austria; 2 Institute of Physical Chemistry and National Center for Computational Design and Discovery of Novel Materials (MARVEL), Department of Chemistry, University of Basel, 4056 Basel, Switzerland; 3 Departments of Chemistry and Physics, University of California, Irvine, California, 92697 USA

**Keywords:** Method development, Materials science, Atomistic models

## Abstract

Over the last decade, we have witnessed the emergence of ever more machine learning applications in all aspects of the chemical sciences. Here, we highlight specific achievements of machine learning models in the field of computational chemistry by considering selected studies of electronic structure, interatomic potentials, and chemical compound space in chronological order.

Accurate solutions of the Schrödinger equation for the electrons in molecules and materials would vastly enhance our capability for chemical discovery, but computational cost makes this prohibitive. Since Dirac first exhorted us to find suitable approximations to bypass this cost^1^, much progress has been made, but much remains out of reach for the foreseeable future. The central promise of machine learning (ML) is that, by exploiting statistical learning of the properties of a few cases, we might leap-frog over the worst bottlenecks in this process.

As visible from the publication record in the field (Fig. 1), over the decade since *Nature Communications* first appeared, machine learning has gained increasing traction in the hard sciences^2^, and has found many applications in atomistic simulation sciences^3^. Here, we focus on the progress achieved in the last decade on three interrelated topics (i) electronic structure theory, broadly defined, (ii) universal force field models, as used for vibrational analysis or molecular dynamics applications, and (iii) first principles-based approaches enabling the exploration of chemical compound space.

**Fig. 1:**
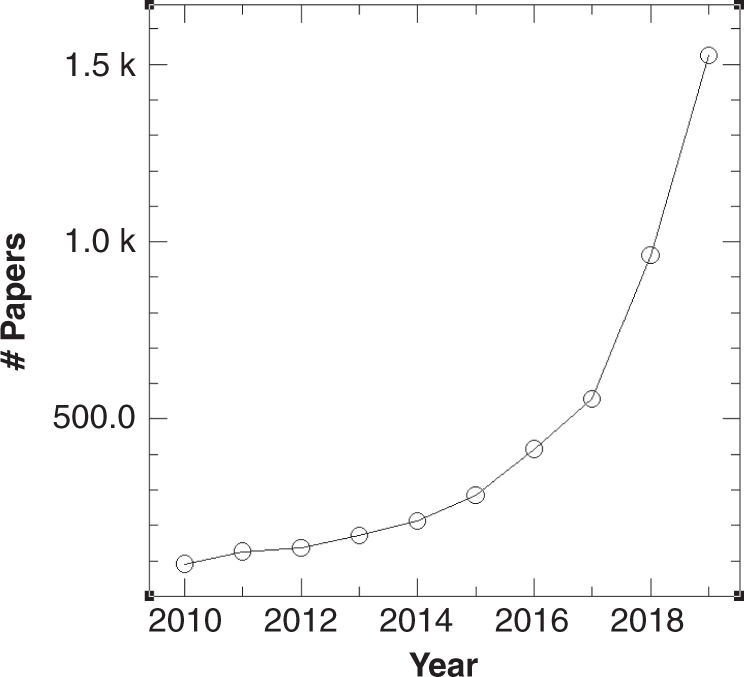
Publications each year from a web of science search with topics of machine learning and either chemistry or materials, July 20, 2020. The average number of citations per article is 12. This updates Fig. 1 of ref. ^30^.

## Basic challenges

The central challenge of Schrödinger space is to use supervised learning from examples to find patterns that either accelerate or improve upon the existing human algorithms behind these technologies. In density functional theory (KS-DFT), this most often means improved approximate functionals; in quantum Monte Carlo (QMC), this is faster ways to find variational wavefunctions; in ab initio quantum chemistry such as coupled cluster considering single, double, and perturbative triple excitations (CCSD(T)), this is learned predictions of wavefunction amplitudes instead of recalculation for every system.

In the condensed phase, molecular dynamics simulations yield a vast amount of useful thermodynamic and kinetic properties. Classical force fields cost little to run, but are often accurate only around the equilibrium. The only first-principles alternative is Kohn–Sham density functional theory (DFT), but its computational cost vastly reduces what is practical. A central challenge of configuration space is therefore to produce energies and forces from a classical potential of accuracies comparable to DFT (at least) via training on DFT-calculated samples, possibly for just one element, but with hundreds to thousands of atoms in unique bonding (and bond-breaking) arrangements.

Finally, the challenge of chemical compound space is to explore all useful combinations of distinct atoms. The number of stable combinations is often astronomical. The central aim is to train on quantum-chemical examples, and create a ML algorithm that can, given a configuration of atoms, generate the atomization energy without running, e.g., a DFT calculation, in order to scan the vast unknown of unsynthesized molecules for desirable functionalities.

These challenges are hierarchical. Progress in creating better density functionals clearly impacts finding accurate forces for molecular dynamics and accurate searching of chemical compound space. Finding a way to learn molecular energies with fewer examples is useful for chemical compound space, but forces would also be needed to run molecular dynamics, and self-consistent densities to run orbital-free DFT. The challenges are also overlapping: improved density functionals may be irrelevant if ML force fields can be trained on CCSD(T) energies and forces.

## Progress with machine learning

### Schrödinger space

Within DFT, the focus is usually on the ever-elusive exchange-correlation (XC) energy^4^, which is needed as a functional of the spin densities. An ‘easier’ target is orbital-free (OF) DFT, which tries to find the kinetic energy of Kohn–Sham electrons, to bypass the need to solve the Kohn-Sham equations. A primary question is: can machines find better density functional approximations than those created by people? Two distinct approaches are to improve the accuracy of existing human-designed approximations or to create entirely new machine-learned approximations that overcome qualitative failures of our present approximations. Often tests are first performed on model systems, and later applied to more realistic first-principles Hamiltonians.

In orbital-free DFT, Snyder et al.^5^ used Kernel-Ridge-Regression (KRR) on a one-dimensional model of a molecule an machine-learned functional for OF DFT that breaks bonds correctly, which has been successively built upon^6^. Brockherde et al.^7^ showed how KRR could be applied by finding densities directly from potentials (the Hohenberg-Kohn map) avoiding functional derivatives. The problem of XC is harder. Nagai et al.^8^ showed that accurate densities of just three small molecules are sufficient to create machine-learned approximations that are comparable to those created by people. In ab initio quantum chemistry, Welborn et al.^9^ have shown how to use features from Hartree-Fock calculations to accurately predict CCSD energies, while an intriguing alternative is to map to spin problems and use a restricted Boltzmann machine^10^. In the last year, two new applications for finding wavefunctions within QMC have appeared^11,12^.

While many avenues are being explored, there is as yet no clearly improved, general-purpose ML-designed density functional, ML-powered QMC, or ML approach to ab initio quantum chemistry available to the general user. But for such a complex problem, progress is measured in decades, and we are reasonably confident that such codes could appear over the next five years.

### Configuration space

Machine learning models for exploring configurational spaces yield rapid force predictions for extended molecular dynamics simulations. While surrogate models of interatomic potentials using neural networks were firmly established before 2010^13^, Csanyi, Bartok and co-workers used KRR in their seminal ’Gaussian-Approximated Potential’ (GAP) method, relying on Gaussian kernel functions and an atom index invariant bispectrum representation^14^. In 2013, the first flavor of the smooth overlap of atomic positions (SOAP) representation for KRR based potentials was published^15^. First stepping stones towards universal force-field, trained ‘on-the-fly’ or throughout the chemical space of molecules displaced along their normal modes, were established in ref. ^16,17^. KRR based force-field models with CCSD(T) accuracy were introduced in 2017^18^, and based on Behler’s atom-centered symmetry function representations in neural network-based potentials tremendous progress was made^19^ enabling Smith et al. to train an Accurate Neural network engIne (ANI) on millions of configurations of tens of thousands of organic molecules distorted along aforementioned normal mode displacements^20^. Impactful applications include KRR potentials used to model challenging processes in ferromagnetic iron^21^, or Weinan E, Car and co-workers using the Summit supercomputer to simulate 100 million atoms of water with ab initio accuracy using convolutional neural networks^22^.

### Chemical compound space

**Fig. 2:**
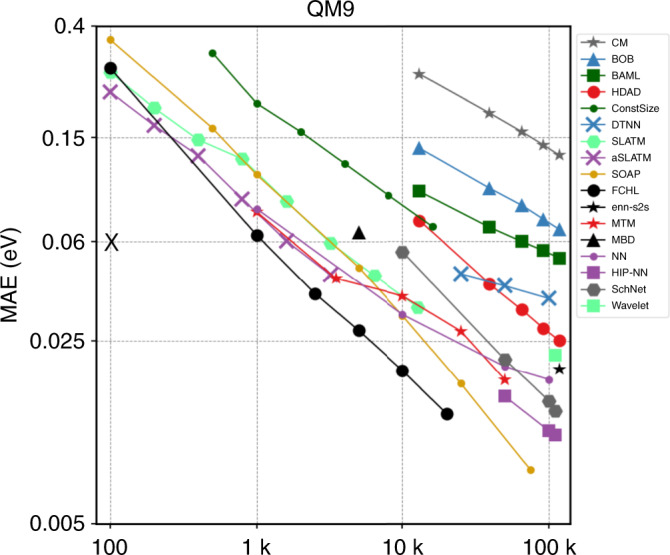
Learning curves of atomization energies of organic molecules, showing out-of-sample prediction error (mean absolute error) decays with increasing number of training molecules drawn at random from QM9 dataset^26^. Models shown differ by representation and architecture. The black X denotes the "QM9 challenge'' of achieving 1 kcal/mol accuracy on the QM9 dataset using only 100 molecules for training^3^. Adapted from ref. ^25^, Springer Nature Limited.

The idea of using machine learning to mine ab initio materials data bases dates back to 2010 in seminal work by Hautier et al.^23^. Starting with the Coulomb-matrix^24^, the development of a selection of ever improved machine learning models (due to improved representations and/or regressor architectures) is exemplified^25^ on atomization energies of the Quantum Mechanics results for organic molecules with up to 9 heavy atoms (QM9) data set^26^, as shown in Fig. 2 “QM9-IPAM-challenge”. Such single-point energy calculations typically dominate the cost of quantum chemistry compute campaigns, and therefore a vital minimal target for surrogate models.

Examples of improvements of understanding compound space include the discovery of an elpasolite crystal containing aluminum atoms with negative oxidation state^27^, polarizability models using tensorial learning^28^, or predicting solvation and acidity in complex mixtures^29^.

## Summary and outlook

Much has happened over the last decade, touching on nearly all aspects of atomistic simulations. Our selection of areas (electronic structure, interatomic potentials, and chemical space) and studies mentioned does not do justice to the overall impact machine learning has had on nearly all branches of the atomistic sciences. Much of the more important work first appeared in rather technical journals such as the *Journal of Chemical Physics* or *Physical Review Letters* and is already heavily cited. More recent advances were published in broader journals such as *Science, PNAS* or *Nature* and *Nature Communications*. Some of the outstanding challenges in the field include (i) improved quantum chemistry methods which can reliably cope with reaction barriers, *d*- and *f*-elements, magnetic and excited states, as well as redox properties of systems in any aggregation state, (ii) extensive high-quality data sets covering many properties over wide swaths of structural and compositional degrees of freedom, and (iii) the removal of hidden and unconscious biases. Extrapolating from the past, the future looks bright: Long-standing problems have been and are being tackled successfully, and new capabilities are always appearing. Likely, the community will soon address challenges that previously were simply considered to be prohibitively complex or demanding, such as automatized experimentation or synthesis of new materials and molecules on demand.
